# Supplementary material for: Skin bacterial community dynamics of hands and forearms before and after military field exercise
Source: Microbiol Spectr. 2025 Apr 15;13(5):e02953-24. doi: 10.1128/spectrum.02953-24 (PMC12054084; doi:10.1128/spectrum.02953-24)
Supplement: Supplemental figures and tables — Figures S1 to S8; Tables S3 to S6. [file spectrum.02953-24-s0001.pdf]

# Supplemental Material

## Supplementary figures

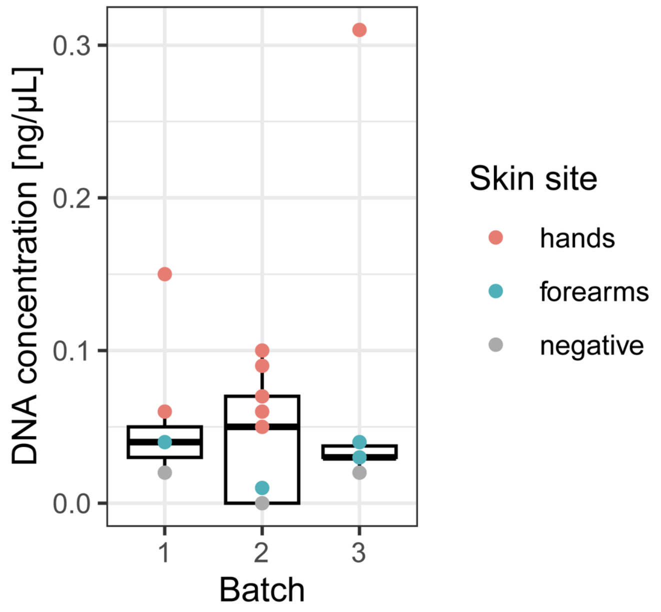


**Figure S1**: **DNA concentration.** Total DNA concentration [ng/µl] in subset samples (*n*=22) taken from hands, forearms, and negative controls (air swabs) per extraction batch. Two outlier values (Z-scores > 3) with concentrations of 0.70 and 0.83 were removed from the dataset before plotting.

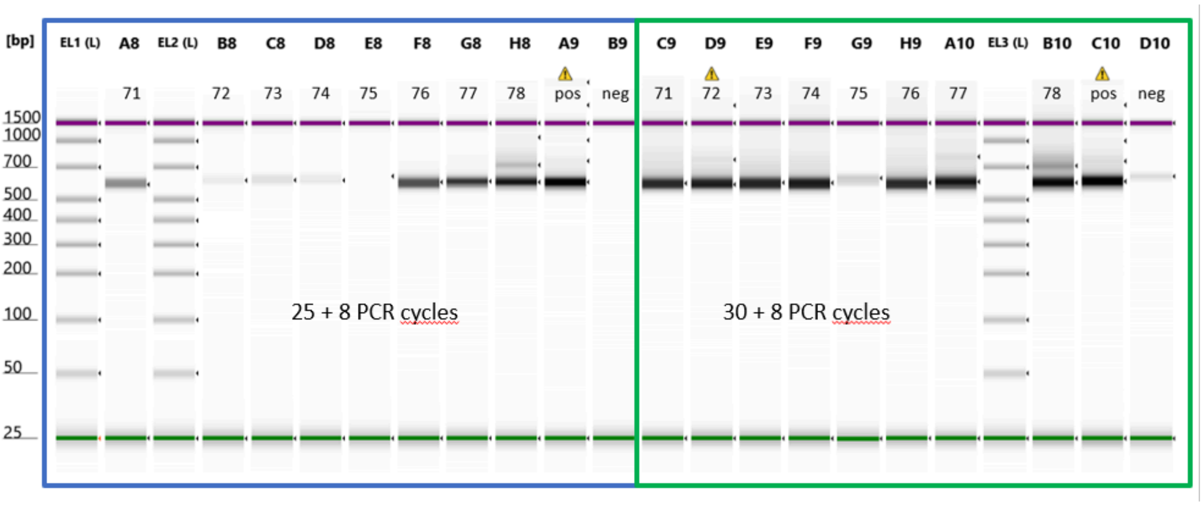


**Figure S2**: **Tests of PCR procedures.** The 2-step PCR amplification was tested using two cycling setups for a subset of skin samples (lanes 71-77), one DNA extraction mock (lane 78), and one positive and negative control. Left: 25 + 8 cycles, right: 30 + 8 cycles.

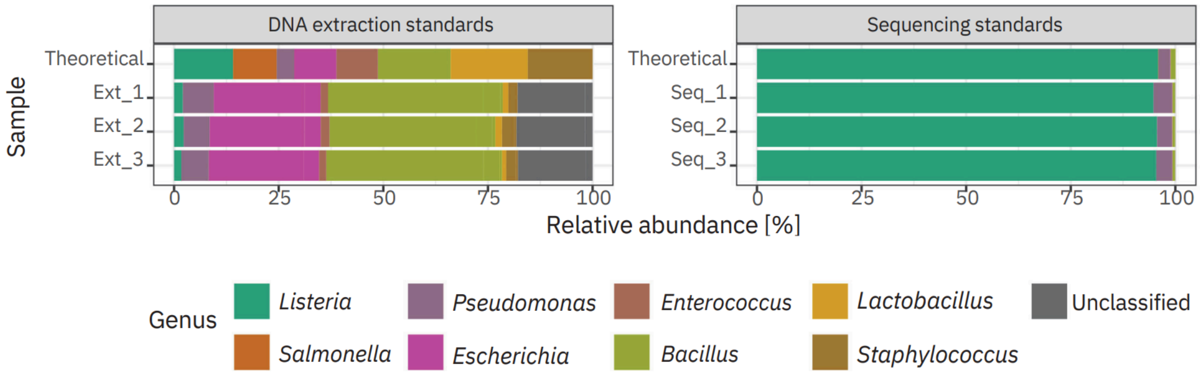


**Figure S3**: **Classification accuracy.** Zymo mock community standards, theoretical vs observed taxonomic distribution (colored by genera). Three replicate samples (one in each extraction batch) for each positive control type. **Left**: Whole-cell standard used in DNA extraction. 7/8 genera present in the mock community were identified. Although *Salmonella* was absent, a related unclassified genus from the Enterobacteriaceae family was detected with an 18 % average abundance. Overrepresentation was noted for *Bacillus* (41.1 %) and *Escherichia* (26.2 %), while *Lactobacillus* (1.28 %), *Staphylococcus* (2.85 %), and *Listeria* (2.02 %) were underrepresented compared to the Zymo standard. **Right**: DNA standards used in library preparation and sequencing. 5/8 genera were identified, with the top 3 genera (*Listeria*, *Pseudomonas*, and *Bacillus*) - accounting for 99.9% of the sequences - having abundances close to theoretical. *Salmonella*, *Staphylococcus*, and *Enterococcus*, with very low theoretical abundances (0.07%, 0.0001%, and 0.000067% respectively), were not detected, though Enterobacteriaceae, likely including *Salmonella*, was identified at 0.0436%.

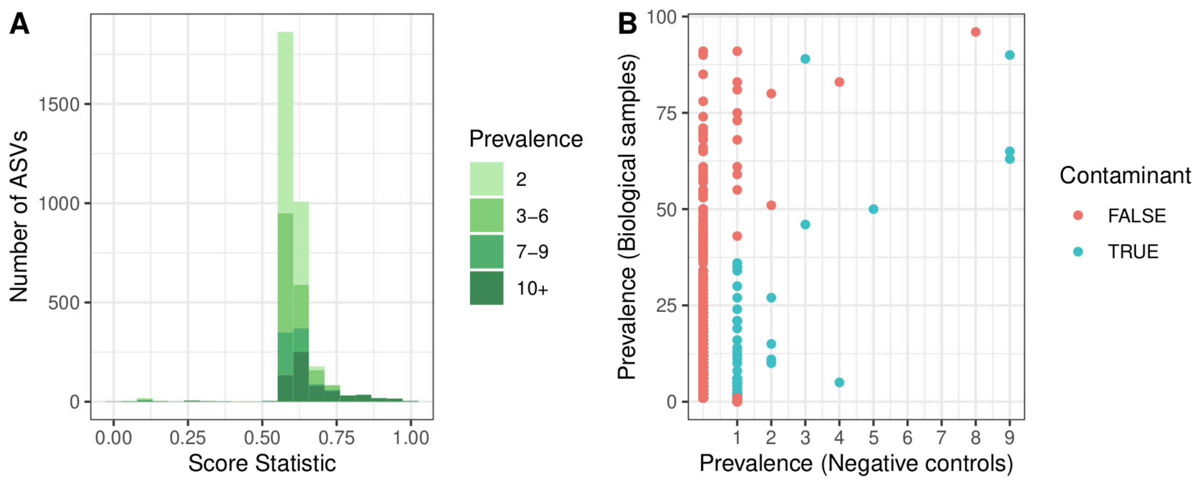


**Figure S4**: ***Decontam()* results. (A)** Distribution of score statistics from *decontam* assigned to each amplicon sequence variant (ASV) based on prevalence (color intensity indicating the total number of samples each ASV was present in (i.e, prevalence). A score < 0.5 indicates a contaminant.

**(B)** Prevalence plot of present/absent ASVs in true samples vs negative controls. Contaminant ASVs identified by *decontam* (with prevalence method) are colored blue.

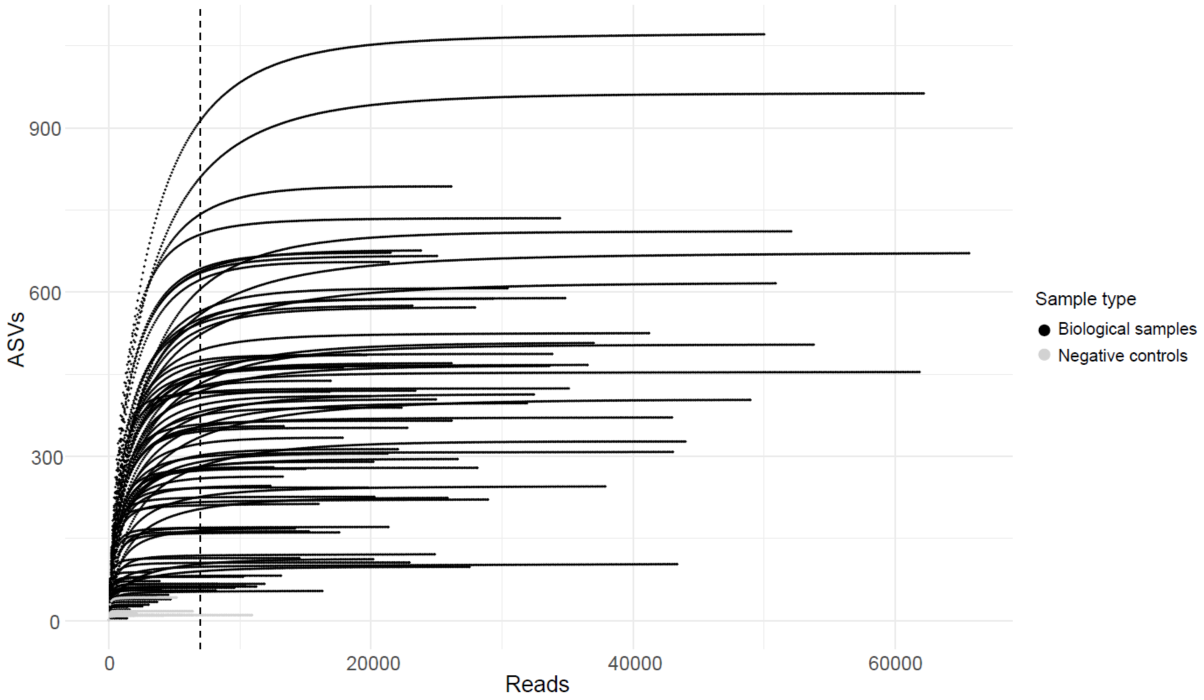


**Figure S5**: **Rarefaction curves.** Number of amplicon sequence variants (ASVs) per sample size (number of trimmed reads) for all biological samples (black, n=96) and negative controls (gray, n=9). The dotted line shows the chosen sampling depth of 7000 reads, keeping most of the diversity of the data (where most samples have reached a plateau of new ASVs per increased depth) and the number of samples.

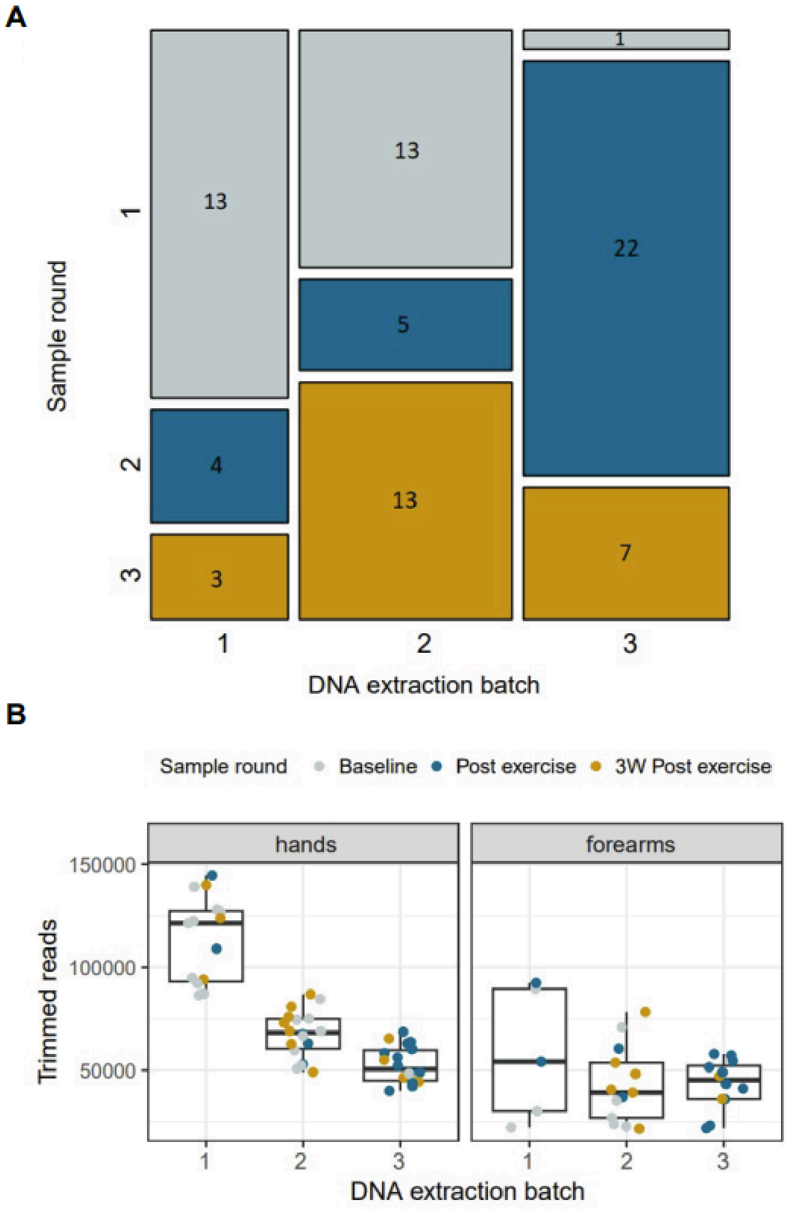


**Figure S6: Batch effects. A)** Contingency table showing the number of samples present in each combination of sample rounds (1: Baseline, 2: Post exercise, 3: 3W Post exercise) and DNA extraction batch (1-3) for all *n*=81 samples (including both skin sites) after rarefaction. **B)** Boxplot showing the number of trimmed reads in each DNA extraction batch, separated by skin site and colored by sample round.

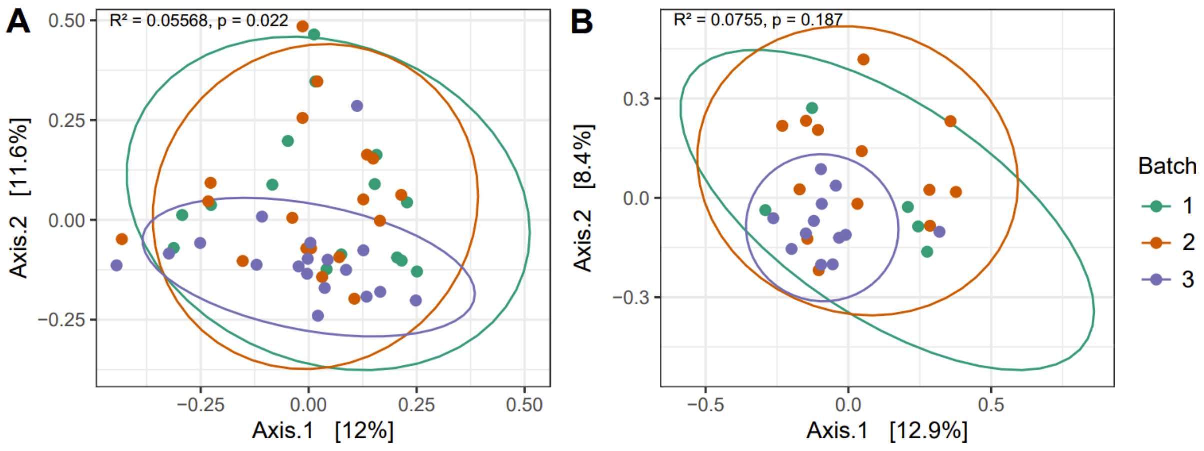


**Figure S7: Beta diversity between batches.** PCoA plot of Bray-Curtis dissimilarity between DNA extraction batches for hand (**A**) and forearm (**B**) samples, with 95 % confidence interval ellipses. Each dot is a sample colored by batch (green = batch 1, orange = batch 2, purple = batch 3). The two axes show the first principal components, explaining the highest percent of variances [denoted in brackets] in the communities. R-squared ($R^2$) represents the proportion of community variance explained by the batch. Significant differences in microbiome composition between batches were found for hand samples, explaining ~6 % of the variance ($p = 0.022$, PERMANOVA). Beta diversity between batches in forearm samples was not significant ($p = 0.187$), not even after ANOSIM testing due to unequal variance between batches ($p = 0.416$).

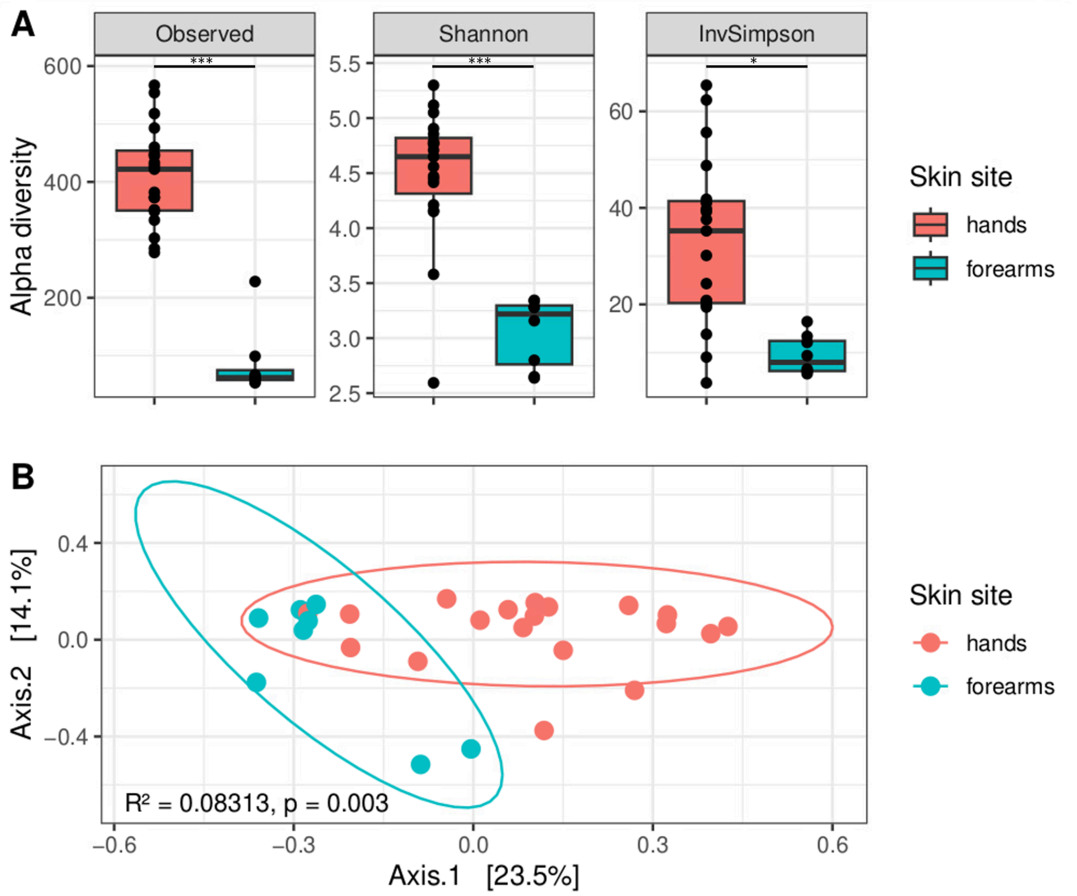


**Figure S8: Baseline differences between hands and forearm samples. (A)** Alpha diversity. Box plots of (unadjusted) bacterial richness (Observed) and evenness (Shannon and Inverse Simpson (InvSimpson) metrics), colored by skin site. Statistical differences between sites found with linear mixed models are marked by asterisks (significance levels: *: 0.05, **: 0.01, ***: 0.001). **(B)** Beta diversity. PCoA plot of Bray-Curtis dissimilarity between hands and forearm samples, with 95 % confidence interval ellipses. The two axes show the principal components explaining the highest percent of variances [denoted in brackets] in the communities. Significant differences in microbiome composition were identified ($p$ = 0.003, PERMANOVA), and the proportion of community variance explained by skin site was $R^2$ = 0.08313.

## Supplementary tables

**Table S3: Linear mixed effects (lme) models and results from statistical comparisons of alpha diversity between sample rounds.** Summary statistics from estimated marginal means (EMMeans) comparison tests of alpha diversity (Shannon and Inverse Simpson) for hands and forearms. Parentheses show upper and lower 95 % confidence levels. P-values are adjusted using the Tukey method for comparing a family of 3 estimates, and those statistically significant ($p<0.05$) are marked in bold.

| | | Hands ($n$ = 51) | | Forearms ($n$ = 30) | |
|---|---|---|---|---|---|
| | | **Shannon** | **Inverse Simpson** | **Shannon** | **Inverse Simpson** |
| **EMMeans** | **Baseline** | 4.40 (4.08-4.73) | 30.7 (21.0 - 40.3) | 3.17 (2.92-3.43) | 12.8 (6.45 - 19.2) |
| | **Post exercise** | 4.70 (4.37-5.03) | 38.2 (21.0 - 40.3) | 4.28 (3.94-4.63) | 29.3 (20.42 - 38.2) |
| | **3W Post exercise** | 4.57 (4.01-5.13) | 43.7 (21.0 - 40.3) | 4.52 (4.13-4.90) | 51.5 (32.77 - 70.2) |
| **Estimated contrasts** | **Exercise effect** | 0.296 ($p$ = 0.273) | 7.54 ($p$ = 0.474) | 1.112 **($p$ = 0.0024)** | 16.5 **($p$ = 0.0445)** |
| | **Total effect** | 0.168 ($p$ = 0.718) | 13.05 ($p$ = 0.170) | 1.346 **($p$ = 0.0002)** | 38.7 **($p$ = 0.0034)** |
| | **3W break effect** | -0.128 ($p$ = 0.865) | 5.51 ($p$ = 0.681) | 0.234 ($p$ = 0.6351) | 22.2 ($p$ = 0.1055) |

**Table S4: PERMANOVA on Bray-curtis dissimilarity (hands and forearms).** Summary statistics from PERMANOVA test on Bray-Curtis dissimilarity values for all (*n* = 81) rarefied samples from hands and forearms combined. P-values are adjusted using the Benjamini-Hochberg method. Organized by $R^2$ in descending order.

| **Factor** | **Df** | **Sum of squares** | **$R^2$** | **F statistic** | **p value** |
|---|---|---|---|---|---|
| Subject | 18 | 7.5548 | 0.33815 | 2.1706 | 0.0013 |
| Sample round | 2 | 1.7209 | 0.07703 | 4.4499 | 0.0013 |
| Skin site | 1 | 0.7172 | 0.03210 | 3.7091 | 0.0013 |
| Extraction batch | 2 | 0.3996 | 0.01789 | 1.0334 | 0.3900 |

**Table S5: PERMANOVA on Bray-curtis dissimilarity (hands only).** Summary statistics from PERMANOVA test on Bray-Curtis dissimilarity values for all (*n* = 51) rarefied hand samples. p values are corrected using the Benjamini-Hochberg method. Organized by $R^2$ in descending order.

| **Factor** | **Df** | **Sum of squares** | **$R^2$** | **F statistic** | **p value** |
|---|---|---|---|---|---|
| Subject | 18 | 5.6371 | 0.44878 | 1.9708 | 0.0015 |
| Sample round | 2 | 1.4559 | 0.11591 | 4.5811 | 0.0015 |
| Extraction batch | 2 | 0.2843 | 0.02264 | 0.8946 | 0.7040 |

**Table S6: PERMANOVA on Bray-curtis dissimilarity (forearms only).** Summary statistics from PERMANOVA test on Bray-Curtis dissimilarity values for all (*n* = 30) rarefied forearm samples. p values are corrected using the Benjamini-Hochberg method. Organized by $R^2$ in descending order.

| **Factor** | **Df** | **Sum of squares** | **$R^2$** | **F statistic** | **p value** |
|---|---|---|---|---|---|
| Subject | 16 | 4.8498 | 0.54424 | 1.1925 | 0.027 |
| Sample round | 2 | 0.8061 | 0.09046 | 1.5857 | 0.009 |
| Extraction batch | 2 | 0.4956 | 0.05562 | 0.9749 | 0.571 |
